# Lipoprotein(a)—60 Years Later—What Do We Know?

**DOI:** 10.3390/cells12202472

**Published:** 2023-10-17

**Authors:** Anna Pasławska, Przemysław J. Tomasik

**Affiliations:** 1 Tuchow Health Center, Medical Hospital Laboratory, Szpitalna St. 1, 33-170 Tuchow, Poland; anna.paslawska@onet.pl; 2 Department of Clinical Biochemistry, Pediatric Institute, College of Medicine, Jagiellonian University, Wielicka St. 265, 30-663 Cracow, Poland

**Keywords:** lipoprotein (a), apolipoprotein (a), cardiovascular disease, coronary heart disease, antisense oligonucleotide, PCSK9 inhibitor

## Abstract

Lipoprotein(a) (Lp(a)) molecule includes two protein components: apolipoprotein(a) and apoB100. The molecule is the main transporter of oxidized phospholipids (OxPL) in plasma. The concentration of this strongly atherogenic lipoprotein is predominantly regulated by the LPA gene expression. Lp(a) is regarded as a risk factor for several cardiovascular diseases. Numerous epidemiological, clinical and in vitro studies showed a strong association between increased Lp(a) and atherosclerotic cardiovascular disease (ASCVD), calcific aortic valve disease/aortic stenosis (CAVD/AS), stroke, heart failure or peripheral arterial disease (PAD). Although there are acknowledged contributions of Lp(a) to the mentioned diseases, clinicians struggle with many inconveniences such as a lack of well-established treatment lowering Lp(a), and common guidelines for diagnosing or assessing cardiovascular risk among both adult and pediatric patients. Lp(a) levels are different with regard to a particular race or ethnicity and might fluctuate during childhood. Furthermore, the lack of standardization of assays is an additional impediment. The review presents the recent knowledge on Lp(a) based on clinical and scientific research, but also highlights relevant aspects of future study directions that would approach more suitable and effective managing risk associated with increased Lp(a), as well as control the Lp(a) levels.

## 1. Introduction

In 1963, 60 years ago, the Norwegian geneticist Kåre Berg identified a unique molecule—lipoprotein(a) (Lp(a)). Research revealed a strong genetic determinant of Lp(a) levels and 11 years after Lp(a) discovery the particles’ elevated values were linked with enhanced coronary heart disease (CHD) incidence. In the 1980s of the last century, some molecular studies allowed the recognition of *LPA* and plasminogen gene (*PLG*) similarity. The essential finding gave a new light to understand the basics of lipoprotein(a) pathophysiology [1]. The multiple studies allowed us to establish Lp(a) as a cardiovascular (CV) risk factor, which increases the risk proportionally to the plasma concentration [2,3,4]. Understanding molecular, biochemical, and structural properties as well as the pathophysiological determinants of the Lp(a) provided crucial insights into the potential Lp(a) risk management strategies. Differences in the particle levels related to ethnicity and apo(a) isoform size are serious challenges to test refinement and standardization. According to the mainly heritable character of Lp(a) levels they are poorly modifiable by diet, behavioral factors, or lifestyle interventions. One of the major purposes of undergoing clinical trials is to establish safe and effective Lp(a) lowering therapy in relation to the improvement of cardiovascular outcomes.

## 2. Lipoprotein(a): Molecular Structure, Genetics, Production, and Catabolism

**Figure 1:**
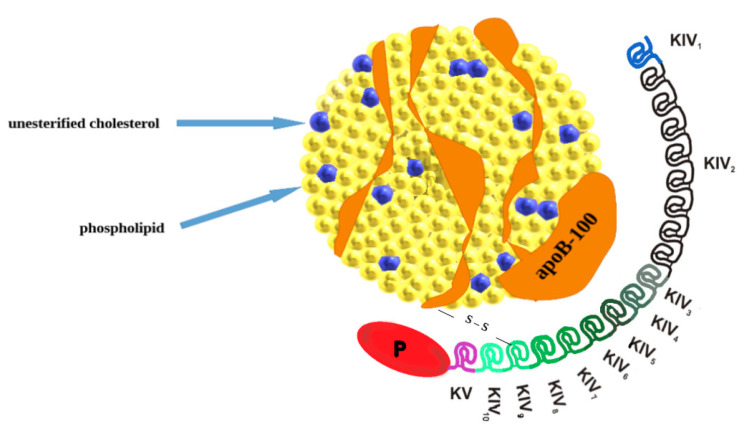
The structure of lipoprotein(a). Lipoprotein(a) consists of two parts: LDL-like particle apolipoprotein B100 (apo-B100) and carried lipids and apolipoprotein(a) [apo(a)] covalently bound by disulfide bonds. Apo(a) is composed of repeating kringle IV (KIV): one copy of both KIV1 and KIV3-10 and variable KIV2 repetition. Additionally, apo(a) is composed of kringle V (KV) and an inactive protease-like domain (P).

Lipoprotein(a) belongs to variants of low-density lipoprotein (LDL) and is composed of two subunits: a variable apolipoprotein a (apo(a)) particle and apolipoprotein B-100 (apoB100) a component of very low-density lipoprotein (VLDL), intermediate-density lipoprotein (IDL), and LDL (Figure 1) [5]. Lp(a) contains apo(a) and apo(b) in a molar ratio of 1:1, covalently bound by a disulfide bridge [6,7,8]. The protein components constitute 30% of the weight of the Lp(a) molecule, allied with associated cholesterol esters (35%), phospholipids (20%), free cholesterol (8%), cholesterol (5%) and triglycerides (2%) [9]. The *LPA* gene is recognized to encode apo(a) with dominant inheritance and the protein component is synthesized in the liver [7]. The *LPA* gene is located at positions 26 and 27 on the long arm of chromosome 6 (6q26–27) [10] and it evolves through replication and modification of the plasminogen gene [9]. The high variability of apo (a) is strictly connected with the protein domain called “kringle” (K). Each kringle (KV, KIV) has a distinctive triple loop structure through six retained cysteine residues that form three disulfide bonds. Apo(a) is composed of a single KV and serine protease-like domain (which is inactive). It also contains 10 subtypes of KIV (KIV1–KIV10) and among them, the KIV2 subtype repeated in many copies (from fewer than 13 to more than 50). The multiple copies are directly responsible for a different size heterogeneity of apo(a) isoforms that are inversely related to Lp(a) levels [10]. High levels of the smaller Lp(a) isoform, with only a few copies of KIV2, are specifically bound to increase cardiovascular risk [11,12,13]. Two single nucleotide polymorphisms (rs10455872 and rs3798220) and a pentanucleotide seem to be responsible for higher Lp(a) concentrations and the possibility of premature CVD [7,14]. Apo(a) is synthesized and secreted from the liver’s hepatocytes in three phases: transcription of the apo (a) gene, translation, and posttranslational modifications required for the apo(a) assembly [9]. The submission of Lp(a) remains unclear, some sources suggest it occurs at the hepatocyte surface [10]. However other studies suppose that the process takes place in plasma, extracellular fluid, or intestinal space [9].

The liver also seems to be the center of Lp(a) catabolism [12]. However, some ‘in vivo’ studies described renal participation in excretion of Lp(a). Apo(a) molecules are excreted by kidneys at a rate of approximately 1–1.5 mg/dL [10]. In reference to the issue, one of the most essential targets of strategy in Lp(a) reducing therapy remains particle clearance.

Due to its particular structure Lp(a) can interact with several receptors. The receptors associated with Lp(a) absorption include LDL receptors (LDLR), VLDL receptors (VLDLR), LDL receptor-related proteins (LRP1 and LRP2), toll-like and scavenger receptors, carbohydrate receptors (lectins) and plasminogen receptors [10,12].

## 3. Pathogenicity of Lp(a)

The physiological function of Lp(a) is still not clear. It could be found in fibrous cap surfaces, in small vessel endothelial cells, and in the extracellular space during the tissue healing process [9]. Therefore, it has considerable participation in tissue regeneration, including wound healing [9,15].

The uniqueness and dualism of Lp(a) particle, based on homology with both plasminogen and LDL-C, are supposed to underly the pathophysiological mechanisms of atherosclerotic cardiovascular disease development [9]. The pathophysiology of Lp(a) is related to atherosclerosis, thrombosis, and inflammation (Table 1). Atherosclerosis is promoted by Lp(a) in two mechanisms [10]. In the first, the particle can infiltrate into the arterial intima and associate elements of the extracellular matrix, stimulating chemotactic activation of monocytes and macrophages, followed by smooth muscle proliferation. Lp(a) binds to macrophages through endocytosis by VLDL-receptors, subsequently, it promotes foam cell formation and cholesterol deposition [16]. There is a strong correlation between Lp(a) levels and oxidized phospholipids (OxPL; transported by apo-B containing lipoproteins) and clinical outcomes. What is more, Lp(a) was eventually recognized as the major carrier of OxPL in plasma [17]. Oxidized phospholipids are involved in monocyte trading into the arterial wall and releasing pro-inflammatory cytokines [9]. Thereby, the atherogenicity of Lp(a) may be connected with the proinflammatory action of OxPL [18]. Also, cytokines induced by Lp(a) promote inflammation. Macrophages can be induced by apo(a) and release interleukin-8, monocyte chemotactic protein, and tumor necrosis factor-α [19].

The third mechanism is based on the similarity of Lp(a) to plasminogen and prothrombotic properties. Lp(a) binds to fibrin and consequently blocks new plasminogen binding. It restricts plasminogen activation as well as its interaction with fibrin [18]. Apo(a) inhibits tissue plasminogen activator (tPA) activation of plasminogen to plasmin and plasminogen binding to fibrin but also promotes increased platelet activity. Hence it leads to thrombosis [20]. Lp(a) is positively correlated with platelet aggregation independent of lipoprotein-associated phospholipase A2 (Lp-PLA2), which may be partly responsible for the atherothrombotic effect of Lp(a). The exact mechanisms demand further investigation [21].

## 4. Lipoprotein(a) and Cardiovascular Disease

Multiple epidemiological surveys have pointed to a connection between cardiovascular diseases and higher levels of lipoprotein(a) (Table 2). One of them, conducted over 11 years with a patient cohort of over 460,000 proved a correlation in atherosclerotic cardiovascular disease risk with elevated Lp(a) levels [22]. This relation was confirmed also in many meta-analyses, genetic studies (GWAS) [23] or Mendelian randomization [24,25]. Recently, researchers found that the risk of myocardial infarction increased by 22% according to a twofold higher concentration of Lp(a) [26]. An observational study that involved over 12,000 patients customized for age, sex, and ethnicity also showed an enhanced risk of MI due to elevated Lp(a) values. The risk was not dependent on other cardiovascular risk factors (smoking, hypertension, and diabetes). The mentioned data indicated a three-fold higher risk of acute myocardial infarction among persons with levels of Lp(a) > 50 mg/dL [27]. Another meta-analysis that gathered more than 126,000 patients presented an enhanced prevalence of coronary heart disease (16%) and stroke (10%) in individuals with higher Lp(a) levels [28]. Increased lipoprotein(a) concentrations determined an elevation (1.97-fold) of risk of cerebrovascular events (both ischemic and hemorrhagic stroke) [29]. A study based on two connected Danish cohorts also confirmed Lp(a) as a stroke determinant [24].

Also, cardiac arrhythmia and valvular diseases are related to higher Lp(a) concentration. According to the research based on data from a biomedical database (UK Biobank) and concerning 435,579 participants, each 50 nmol/L (23 mg/dL) increase in Lp(a) was associated with an elevated risk of incidence of atrial fibrillation by 3% [29]. Conversely, previous studies indicated an inverse relationship (the higher the Lp(a) level the lower the risk of AF incidence) [30,31,32]. Two extensive, Danish cohort studies (the Copenhagen City Heart Study and the Copenhagen General Population Study) lasting 20 years (77,000 patients) confirmed the enhanced danger of aortic valve stenosis (AVS) among individuals that presented higher Lp(a) values (three-fold elevated danger when concentration >90 mg/dL) [33]. AVS progression as well as the necessity of a previous reaction in those with increased lipoprotein(a) level was also acknowledged in post hoc analysis of the Astronomer trial [34]. However, recently published data specified Lp(a) as a factor involved in the beginning of AVS and excluded its participation in the disease progress [35].

Moreover, Lp(a) concentration correlates with Peripheral Arterial Disease (PAD). Elevated plasma Lp(a) levels enhance the incidence of PAD and hospitalization related to the disease. Higher levels of Lp(a) are associated with multiple peripheral artery revascularization, and serious dismemberment among patients suffering from PAD or new peripheral lesions. Lp(a) concentration may be predictive of atherosclerosis correlated with cardiovascular events, including Critical Limb Ischemia or other lower-limb events among patients with PAD [36,37].

In addition to the mentioned cardiovascular diseases, there is an association between plasma Lp(a) levels and advanced fibrosis in patients with NAFLD (nonalcoholic fatty liver disease). Patients with low plasma Lp(a) concentration presented insulin resistance, elevated transaminase, and enhanced risk of developing severe fibrosis and cirrhosis. Furthermore, Lp(a) level in combination with transaminases may represent a novel noninvasive predictive biomarker of advanced fibrosis in patients with NAFLD [38].

## 5. Ethnicity and Non-Genetic Factors Influencing Lp(a) Concentrations

Lp(a) plasma concentration is mainly genetically regulated and varies by ethnicity as well as apo(a) isoform size [39]. Africans and African descendants have the highest Lp(a) level compared with other ethnicities (subsequently South Asians, Whites, Hispanics, and East Asians). Racial differences are partly conditioned by apo(a) size and LPA gene polymorphisms.

There are some non-genetic factors that can also influence levels of Lp(a). Taking diet into consideration, saturated fats replaced with carbohydrate or unsaturated fats increase Lp(a) level by 8–20% [19]. Meanwhile, a low-carbohydrate or high-saturated fat diet decreases the level approx.15% [40]. The noticeable lowering effect on Lp(a) levels was observed in connection with flaxseed or walnut supplementation [19,41]. Lp(a) levels are not strictly associated with age or gender though some studies showed higher levels in females than males [23]. Endogenous sex hormones have no or minor association contrary to postmenopausal hormone replacement therapy (HRT) that decreases Lp(a) levels by 20–25% [42]. Lp(a) levels increase one to twofold in pregnancy and return to a normal level after delivery [10]. Treatment of overt and subclinical hypothyroidism reduces Lp(a) by 5–20% [43] while growth hormone replacement therapy causes an increase in its concentration by 25–100% [44]. Physical activity and exercise have no or minimal impact on Lp(a) [42]. Acute inflammatory illnesses, like sepsis or inflammatory bowel disease, increase Lp(a) concentration along with α 1 antitrypsin, IL-6, and CRP [9] and the level is normalized with recovery. Elevated Lp(a) accompanies kidney impairment starting from the early stages of their disability and in non-nephrotic kidney disease (NNKD) its levels have an inverse relation with kidney function [42]. Kidney transplantation normalizes the Lp(a) levels to baseline [45]. In most forms of liver disease with hepatocellular damage Lp(a) levels are reduced in parallel with the disease progression (over 40% reduction in hepatitis). Though Lp(a) concentration increases twofold during antiviral treatment [46].

## 6. Lp(a) in Children and Youth

Right after birth, Lp(a) values remain low which refers to incomplete LPA gene expression [47]. According to some sources, Lp(a) level is determined approximately second year of life [47,48,49]. A child with elevated Lp(a) level is to become certainly an adult with increased Lp(a). However, other studies showed that Lp(a) concentrations increase during childhood, and they fluctuate during growing up [50]. It is supposed that hormonal changes related to maturation or proinflammatory factors which may influence hormones are responsible for this phenomenon [51]. The mentioned situation undermined the assumption that one determination of Lp(a) in childhood is sufficient. As the values of Lp(a) vary in childhood, there appears rationale for retrying the measurement in young adults to estimate the level and related risks more accurately [50].

Mostly, young patients with elevated Lp(a) concentrations, have no clinical symptoms in the youth [47]. However, in some pediatric patients, especially in those burdened with family medical history (e.g., obesity, diabetes, hypertension, dyslipidemia) increased Lp(a) concentrations, similarly to adults, are bound to an elevated risk of myocardial infarction, ischemic stroke or peripheral venous thrombosis [47,52,53]. The American Heart Association released in 2019 a scientific claim that highlighted the evident role of Lp(a) screening among pediatric patients with familial hypercholesterolemia to specify those at ‘high-risk’ of premature cardiovascular disease [54]. Young individuals with diabetes mellitus present an association between Lp(a) and LDL cholesterol concentration; however, this relation is more significant in Black children than in White young diabetics [55].

These data suggest that Lp(a) should be tested in children, at least in those with individual or familial risk of CVD. Health-promoting behaviors (e.g., physical activity, diet) and finally considering lipid-lowering treatment, enable young patients to prevent premature ASCVD and diminish those risks in later life. Therapies directly lowering Lp(a) are currently in clinical trials in adults [56,57] so probably with some delay, the dedicated treatment will be available for pediatric patients [57].

Beyond specifying treatment that will be suitable, the Lp(a) thresholds within different age and race groups should be established as well as guidelines for testing—population or groups of risk screening.

## 7. Measurement of Lipoprotein(a), Cut-Off Values and Risk Stratification

The unique structure of the Lp(a) particle implicates complications with the accuracy of its measurement. Many repetitive KIV repeats in apo(a) determine Lp(a) size heterogeneity which makes the standardization very challenging [33]. The main aspects of Lp(a) measurement concern the isoform-sensitivity of measurement systems, assay calibrators, and the type of antibodies used for the measurement [58]. There were observed cross-reactions of anti-apo(a) antibodies with repetitive KIV2. As a result, persons with large isoforms obtain falsely high results of Lp(a) concentration in some methods [33].

Lp(a) expressed in mass units (mg/dL) includes all particle constituents (apo(a), apoB-100, cholesterol, cholesteryl esters, phospholipids, and triglycerides) [7,16]. Lp(a) measurements can be also expressed in molar concentration (nmol/L) which is more indicative of particle numbers. Conversion between mass and molar units is not recommended due to inaccuracy [16]. Standardization using a single calibrator is unreliable because of the diversity and two isoform sizes occurrence (most kits now use 5-point calibration). According to that, lipoprotein(a) values ought to be expressed in nmol/L and measured using isoform-insensitive methods based on appropriate antibodies with calibrators traceable to the WHO/IFCC reference material [16]. The assay that relies on antibodies binding KIV9 (i.e., ELISA), the unique nonrepeating kringle IV subtype, is recognized to be the gold standard and preferred to evaluate Lp(a) [9]. To standardize Lp(a) measuring, a method of mass spectrometry has been recently validated. The method allows us to avoid problems with size polymorphisms as it is based on quantifying particular peptides not found in the KIV2 region [59].

The essential aspect of Lp(a) analysis of obtained results are right normal values. Lp(a) has asymmetric distribution among different races that demand to determine suitable cut-offs for particular populations. According to the American Heart Association/American College of Cardiology (AHA/ACC), and the European Society of Cardiology (ESC) the threshold value of 50 mg/dL (>125 nmol/L) is related to increased cardiovascular risk. Nevertheless, starting from over 30 mg/dL (75 nmol/L) the risk is reported to be progressive [15].

HEART UK consensus statement has suggested classified categories based on Lp(a) centiles that deal with cardiovascular disease risk [16]:

- 32–90 nmol/L—little chance of cardiovascular disease;
- 90–200 nmol/L—modest chance;
- 200–400 nmol/L—large chance;
- >400 nmol/L—very high chance.

Following the HEART UK indications lipoprotein(a) levels should be measured once, unless a secondary cause is suspected, or special lowering treatment is implemented. Moreover, the Lp(a) levels should be measured in persons with (a) a personal or family history of premature atherosclerotic cardiovascular disease (<60 years of age), (b) first-degree relatives with raised serum Lp(a) levels (>200 nmol/L), (c) familial hypercholesterolemia (FH), or other genetic dyslipidemias, (d) calcific aortic valve stenosis, (e) a borderline increased (but <15%) 10-year risk of a cardiovascular event [16].

The American College of Cardiology and Canadian Cardiovascular Society guidelines indicate the reasonableness of Lp(a) measurement in persons with a family history of premature cardiovascular disease [60,61]. It is also regarded to be useful in adults 40–75 years old with no diabetes mellitus and intermediate risk for ASCVD. ESC in turn, has recommended measuring Lp(a) in the general population minimum once over a lifespan in order to specify patients with strongly elevated Lp(a) values > 180 mg/dL (>430 nmol/L) [15] (Table 3).

## 8. Treatment of Increased Lp(a)

Although there is an established connection between increased levels of Lp(a) and cardiovascular diseases, there are no direct recommendations on routine therapy lowering Lp(a) [15,62,63]. The reason is the lack of specific Lp(a) lowering treatment. Despite a favorable impact on lipid profile, a low-fat diet as well as physical activity have modest effects on Lp(a) levels [23]. So, the main targets for the therapy of persons with heightened Lp(a) levels are the other cardiovascular risk factors [64]. They involve lifestyle and dietary adjustment as well as treatment of hypertension or diabetes and cholesterol-lowering therapy, if necessary. The target level of LDL cholesterol is determined individually, but in patients at increased risk is recommended to reduce it to 2.5–3 mmol/L [65]. Patients that have high to very high CV risk have LDL targets specified for particular conditions (LDL target for atherosclerotic disease, familial hypercholesterolemia, or diabetes) [66].

Clinically available and the most effective intervention for Lp(a) decreasing is lipoprotein apheresis [67,68]. According to the Food and Drug Administration, patients qualified for apheresis present functional familial hypercholesterolemia, coronary artery disease and LDL-C >100 mg/dL and Lp(a) > 60 mg/dL. The medical procedure is typically performed every two weeks (in some countries weekly). During the one course (3–4 h) the Lp(a) concentration can be lowered by 50% to 85%. Additionally, lipoprotein apheresis reduces LDL levels by 60% to 85% [69]. It is expected that such Lp(a) lowering reduces the risk of ASCVD events.

The impact of statin therapy on lipoprotein(a) concentration remains contested. Independent surveys presented enhanced Lp(a) values among patients treated with statins (probably in those with small apo(a) phenotype) [70,71]. Other data, from the JUPITER study, revealed no change in Lp(a) with rosuvastatin treatment [33]. Nevertheless, despite minimal Lp(a)-lowering efficacy, statins reduce the overall cardiovascular risk (by LDL-C and apoB lowering) and decrease the risk of CV incidents [62].

Niacin may decrease Lp(a) levels by up to 40% in individuals with high Lp(a) values [72]. The main effect is based on lowering apo(a) production [73]. Besides the reduction of Lp(a), niacin lowers LDL-C and triglycerides, and increase HDL level. However, the therapy does not implicate a decrease in cardiovascular events. Furthermore, the combined therapy containing niacin and statins is limited by many side effects (e.g., infections and bleeding, diabetes onset, gastrointestinal discomfort) [56].

Another non-specific Lp(a) lowering therapy includes anti-PCSK9 antibodies (inhibitors of Proprotein Convertase Subtilisin/Kexin type 9). The treatment is routinely designed for LDL cholesterol reduction. Nevertheless, recent remarks suggested that PCSK9 inhibitors affect Lp(a) level in accordance with limiting its production and heightening its clearance. It is necessary to mention that the issue of the precise mechanism needs to be clarified [74]. The FOURIER and ODYSSEY studies analyzed evolocumab—reduced Lp(a) by 29.5% [23,74], and either alirocumab—decreased Lp(a) by 23.5% [63,75]. These drug-mediated Lp(a) lowering were independently associated with cardiovascular events reduction [75].

Also, inclirisan, another drug based on small-interfering RNA (siRNA) technology inhibits the hepatic synthesis of PCSK9 and influences the Lp(a) level. Following trials, ORION 10 and ORION 11, presented their influence on the overall reduction of Lp(a) by ≈20% [76,77]. In fact, all PCSK9 inhibitors’ target remains the reduction of LDL cholesterol with slight effects on Lp(a) values [23].

There are some new, up today experimental therapies, that selectively reduce the lipoprotein(a) levels. There are two attempts—using apo(a) antisense oligonucleotide (ASO) and those based on siRNA technology [78]. Both classes of drugs are administered subcutaneously. The ASO class involved IONIS-APO(a)-Rx and its improvement Pelacarsen (TQJ230 or AKCEA-APO(a)-LRx). The latter has good tolerance and around 80% efficacy in Lp(a) lowering [79]. They also reduce the expression of the pro-inflammatory gene in circulating monocytes and restrict their transendothelial migration capability [80]. The ongoing trial (HORIZON, NCT04023552) evaluates the influence of Lp(a) reducing using Pelacarsen on ASCVD outcomes among over 8000 patients and is planned to be completed in the first half of 2025 [23]. Also, three drugs of the siRNA class are under assessment. They are designed to suppress *LPA* mRNA hepatocellular translation. Olpasiran (AMG890) has shown decreasing Lp(a) level properties (by 80–94%) in patients with initial 70 nmol/L–199 nmol/L Lp(a) values. This effect was a little bit weaker among persons presenting Lp(a) levels over 200 nmol/L [81]. Some official results of the second phase research with Olpasiran (OCEAN(a)-DOSE; NCT04270760) are to be published. Nevertheless, Lp(a) reducing efficacy has been confirmed in a recent report (about 98% for a dose of 225 mg every 3 or 6 months) [82]. The study using the next siRNA-based drug (SLN360) has completed phase 1 and entered phase 2. The ultimate dosage (600 mg) was related to Lp(a) concentration reducing by 20% with at least 5 months of persistence [83]. The last siRNA class medicament (LY3819469) has already completed 1 phase trial. All these potential novel RNA-targeting drugs have so far satisfactory pharmacokinetic properties, and stable and consistently high, dose-dependent efficacy in decreasing Lp(a). The mentioned therapies presented no significant safety concerns [84]. Some commercially accessible (non-specific) and experimental therapies (specific) lowering Lp(a) concentration have been presented in (Table 4).

Although lifestyle interventions may not directly influence Lp(a) levels or change them slightly, a healthy diet and/or regular physical activity are recommended for cardiovascular disease prevention. The mentioned lifestyle factors can improve the lipid profile. Decreasing LDL-C (suitable diet) and increasing HDL-C (physical activity) values is highly recommended to mitigate the synergistic risk of high LDL and Lp(a) concentrations and ought to be the basis of elevated Lp(a) cardiovascular prophylaxis.

## 9. Conclusions

Lp(a) is a genetically determined, prevalent risk factor for atherosclerotic cardiovascular disease, e.g., aortic valve stenosis, myocardial infarction, or ischemic stroke. Lp(a) reduction is expected to improve cardiovascular outcomes concurrently. The methods of measurement require standardization or at least harmonization, as well as method cut-off values for subsequent ethnic groups. The management of patients with elevated lipoprotein(a) values (>90 nmol/L) should involve reducing overall atherosclerotic risk and persistent controlling of hyperlipidemia. The Lp(a) levels in children are not well established. So, pediatric reference ranges in different race groups should be determined as well as thresholds for pediatric patients at risk of premature CVD (familiar hypercholesterolemia, and some other dyslipidemias, diabetes mellitus, and obesity). Also, guidelines for Lp(a) testing in childhood—populations or groups of risk screening should be prepared. Most of the available therapies are standard lipid-lowering treatments; however, there are several encouraging agents in the last stages of clinical trials. Currently experimental, ASO inhibiting apo(a) or siRNA inhibiting apo(a), may become ultimately specific, directly lowering Lp(a) medicines used in daily, clinical practice. The lack of definitive proof that available therapies not only reduce Lp(a) level but also demonstrate a positive effect on CVD outcomes determines further research.

## Figures and Tables

**Table 1:** Proatherogenic and prothrombotic mechanisms of action of Lp(a).

Proatherogenic and Proinflammatory Properties of Lp(a)	Prothrombotic Properties of Lp(a)
↑ Oxidized phospholipids ↑ Foam cell formation ↑ Endothelial dysfunction ↑ Smooth muscle cell proliferation ↑ Chemoattraction of monocytes ↑ Inflammation of the arterial wall (IL-8, monocyte chemotactic protein, TNF-α)	↓ Plasminogen activation ↓ Fibrinolysis ↓ Tissue factor pathway inhibitor ↓ Clot permeability ↑ Platelet response ↑ Plasminogen activator inhibitor-1 (PAI-1)

**Table 2:** Epidemiological research implying a causal link of Lp(a) with CVD.

Study	Group of Patients	Outcome	Implications
National Biobank [22] Lipoprotein(a) levels and the MI risk among different ethnicities, INTERHEART study [2] Lipoprotein(a) values vs. CV risk, meta-analysis [29] The Copenhagen City Heart study [24] The Copenhagen General Population study [24]	460,506 12,943 126,634 10,813 49,699	Prevalence of ASCVD Prevalence of MI Prevalence of CVA and CHD CV results based on register AVS prevalence	Elevated risk of ASCVD incidents Increased risk of MI Increased association of Lp(a) with CVA and CHD Elevated incidence of MI and AVS Three fold elevated AVS risk when Lp(a) > 90 mg/dL

Abbreviations: ASCVD—Atherosclerotic Cardiovascular Disease; MI—Myocardial Infarction; CVA—Cerebrovascular accident; CHD—Coronary Heart Disease; CV—cardiovascular; AVS—Aortic Valve Stenosis.

**Table 3:** Recommendations for lipoprotein(a) population screening.

Recommendation	Currently Applying Screening Guidelines
2018 ACC/AHA (American College of Cardiology/American Heart Association) Cholesterol Guidelines [60] 2019 ESC/EAS (European Society of Cardiology /European Atherosclerosis Society) Dyslipidemia Guidelines [15] HEART UK Consensus Statement [16] 2021 Canadian Cardiovascular Society Guidelines [61]	- No specific screening recommendation; increased Lp(a) (≥50 mg/dL or 125 nmol/L) is regarded as a “risk enhancing” factor. - Lp(a) measurement in persons with family history of premature cardiovascular disease. - Lp(a) measurement is useful in adults 40–75 years old with no diabetes mellitus and with 10-year ASCVD risk of >5–19.9%. - Lp(a) measurement at least once in adult’s lifetime to identify those with very high Lp(a) levels > 180 mg/dL (>430 nmol/L). - Account Lp(a) measurement in selected individuals with family history of premature coronary artery disease or elevated Lp(a). - To reclassificate risk in patients that are at borderline between moderate to high risk of CVD. - Measurement once, unless a secondary cause is suspected or special lowering treatment is implemented. - First degree relatives with raised serum Lp(a) levels (>200 nmol/L). - Personal or family history of premature atherosclerotic cardiovascular disease (<60 years of age). - Calcific aortic valve stenosis. - A borderline increased (but <15%) 10-year risk of a cardiovascular event (reclassification). - Familial hypercholesterolemia (FH), or other genetic dyslipidemias. - Family history of premature ASCVD.

**Table 4:** Non-specific and specific (experimental) therapies for Lipoprotein(a) decrease.

Drug/Intervention	Mechanism of Action	Lp(a) Level Decrease	CV Risk Reduction
**Non-specific therapies**			
Lipoprotein apheresis [67,68,69]	2–3-h procedure; plasma exchange; removes LDL-c, VLDL, apoB containing particles (Lp(a))	>50%	Yes
Statins [71,72]	Inhibition of HMG-CoA reductase enzyme	Conflicting results	Yes
Niacin [56,72,73]	Inhibits triglycerides synthesis	20–40%	No
Evolocumab [23,74]	Anti-PCSK9 antibodies	29.50%	Yes
Alirocumab [63,75]	Anti-PCSK9 antibodies	23.50%	Yes
Inclisiran [76,77]	siRNA inhibiting PCSK9	≈20%	No
**Specific (experimental) therapies**			
Pelacarsen [79,80]	ASO inhibiting apo(a)	≈80%	Experimental phase (phase 3 ongoing)
Olpasiran [81,82]	siRNA inhibiting apo(a)	80–98%	Experimental phase (phase 3 ongoing)
SLN360 [83]	siRNA inhibiting apo(a)	98.00%	Experimental phase (phase 2 ongoing)
LY3819469 [84]	siRNA inhibiting apo(a)	unknown	Experimental phase (phase 2 registered)

## Data Availability

Not applicable.
